# Small dense LDL particles - a predictor of coronary artery disease evaluated by invasive and CT-based techniques: a case-control study

**DOI:** 10.1186/1476-511X-10-21

**Published:** 2011-01-25

**Authors:** Anne P Toft-Petersen, Hans H Tilsted, Jens Aarøe, Klaus Rasmussen, Thorkil Christensen, Bruce A Griffin, Inge V Aardestrup, Annette Andreasen, Erik B Schmidt

**Affiliations:** 1 Department of Cardiology, Center for Cardiovascular Research, Aalborg Hospital, Aarhus University Hospital, Aalborg, Denmark; 2 Department of Radiology, Center for Cardiovascular Research, Aalborg Hospital, Aarhus University Hospital, Aalborg, Denmark; 3 Faculty of Health & Medical Sciences, University of Surrey, Guildford, Surrey, GU2 7XH UK

## Abstract

**Background:**

Coronary angiography is the current standard method to evaluate coronary atherosclerosis in patients with suspected angina pectoris, but non-invasive CT scanning of the coronaries are increasingly used for the same purpose.

Low-density lipoprotein (LDL) cholesterol and other lipid and lipoprotein variables are major risk factors for coronary artery disease. Small dense LDL particles may be of particular importance, but clinical studies evaluating their predictive value for coronary atherosclerosis are few.

**Methods:**

We performed a study of 194 consecutive patients with chest pain, a priori considered of low to intermediate risk for significant coronary stenosis (>50% lumen obstruction) who were referred for elective coronary angiography. Plasma lipids and lipoproteins were measured including the subtype pattern of LDL particles, and all patients were examined by coronary CT scanning before coronary angiography.

**Results:**

The proportion of small dense LDL was a strong univariate predictor of significant coronary artery stenosis evaluated by both methods. After adjustment for age, gender, smoking, and waist circumference only results obtained by traditional coronary angiography remained statistically significant.

**Conclusion:**

Small dense LDL particles may add to risk stratification of patients with suspected angina pectoris.

## Introduction

Coronary artery disease (CAD) is a common cause of morbidity and mortality in the industrialised world [1]. The diagnosis of coronary atherosclerosis is usually made by invasive coronary angiography (CAG) during which percutaneous coronary intervention (PCI) procedures can be applied simultaneously. CAG is invasive, exposes the patient to a moderate amount of radiation and iodinated contrast agent, and depicts only atherosclerotic lesions that bulge into the lumen. Among the currently available non-invasive alternatives, CT-based coronary angiography (CT CAG) provides a view of the vessel lumen as well as the architecture of the vessel wall. Additionally, the degree of calcification, which has been shown to be a prognostic marker for CAD [2], can be accessed. Disadvantages of CT CAG include the relatively large dose of radiation, the inability to perform intervention, and inconclusive scans. Equipment for CT CAG is, however, being improved, and presumably this will reduce the failure rate and lead to a lower dose of radiation. CT CAG, measured with invasive CAG as the golden standard, offers a high sensitivity for coronary stenoses, whereas the specificity is moderate [3]. It has therefore been argued that patients at low or moderate risk of CAD should undergo a "screening" CT CAG that could select patients for possible intervention.

An elevated concentration of LDL cholesterol is a major risk factor for CAD [4-6]. However, LDL consists of a heterogeneous spectrum of particles with highly variable atherogenic potential [7]. Small dense LDL particles (sdLDL) are believed to be particularly atherogenic due to increased susceptibility to oxidation [8,9], high endothelial permeability [10], decreased LDL receptor affinity [11], and an increased interaction with matrix components [5].

The main hypothesis to be tested was that sdLDL might be a better predictor of coronary atherosclerosis than standard lipids and lipoproteins. Furthermore, we evaluated levels of apoprotein (apo) B and lipoprotein (a) (Lp(a)) in patients with chest pain considered at low or intermediate risk for CAD and investigated by CAG and CT CAG.

## Methods

We performed a case-control study of 194 consecutive patients with low or intermediate risk of CAD, referred for elective CAG (invasive CAG) at Aalborg Hospital between June 2007 and December 2008. Causes of referral were angina pectoris or angina equivalent symptoms. Based on information of previous history, current symptoms, and risk factors patients were categorized clinically as having a high risk or a low to intermediate risk of CAD. We carried out a CT CAG as well as an invasive CAG in all patients. Based on this, we grouped the patients in two parallel analyses, i.e. CAD/no CAD on CAG and CAD/no CAD on CT CAG, thus allowing parallel comparisons of sdLDL as a risk factor for CAD measured by different, yet clinically relevant, diagnostic modalities. Patients underwent a CT CAG and later, but within the same week, an invasive CAG, unless they met any of the following exclusion criteria:

• A prior diagnosis of CAD, verified by:

○ elevated myocardial enzymes

○ signs of myocardial ischemia in ECG either spontaneously or during a stress test

○ significant stenoses in the coronary arteries demonstrated in an earlier invasive CAG

○ ischemia demonstrated by myocardial perfusion scintigraphy or stress echocardiography

• Invasive CAG referral due to defect cardiac valves, cardiomyopathy, or cardiac arrhythmias

• Elevated serum creatinine

• Diabetes mellitus

• Known intolerance to contrast agents

• Inability to hold the breath for at least 10 s in connection with the CT CAG

• Contraindications against intravenously administered β-adrenoceptor-antagonists

• Age less than 40 years

• Lack of contraception in premenopausal women

Patient characteristics are given in Table 1. Of the 194 patients enrolled, 3 dropped out before blood sampling, and 13 were excluded as diabetics. Of the 178 completing, non-diabetic participants, 16 had a technically unacceptable CT CAG and were excluded from this part of the study. Three patients had serum triglycerides ≥5 mmol/L, and as this interfered with the calculation of LDL cholesterol, they were excluded from the LDL cholesterol comparisons as well as the adjusted analyses.

**Table 1:** Basic characteristics of the patients included in the study

Variable	n = 178
Age (mean and SD)	62.4 ± 9.6

Gender (male) (%)	49.4%

Family disposition to ischemic heart disease (%)	52.8%

Current smoking (%)	21.3%

Lipid-lowering medication (%)	59.0%

Antihypertensive medication (%)	67.4%

Systolic blood pressure (mean and SD)	146.7 ± 20.2

Diastolic blood pressure (mean and SD)	79.3 ± 10.5

BMI (kg/m^2^) (mean and SD)	26.9 4.0

Waist circumference (cm) (mean and SD)	96.3 11.8

Triglycerides (mmol/L) (mean and SD)	1.5 1.0

HDL cholesterol (mmol/L serum) (mean and SD)	1.6 0.5

LDL cholesterol (mmol/L serum) (mean and SD) (n = 175)	2.8 0.9

High sensitive CRP (mean and SD)	2.8 4.7

ApoB (g/L) (mean and SD)	0.9 0.2

Lp(a) (arb. units) (mean and SD)	361.1 435.5

### CAG and CT CAG

On the day of the CT scan, venous blood samples (50 mL) were drawn. Serum lipids (total cholesterol, high-density lipoprotein (HDL) cholesterol, and triglycerides) were measured by routine methods at the central laboratory, and LDL cholesterol was calculated according to the Friedewald formula. Serum samples for the measurement of apo B, Lp(a), and highly sensitive (hs)CRP were frozen at -80°C and analysed after all patients had been included. Apo B was measured using antibody from DAKO, Glostrup, Denmark, on an Advia 1650 from Bayer Diagnostics, NY, US. Lp(a) was measured using an ELISA kit from Mercodia, Uppsala, Sweden, while hsCRP was measured by an immunoturbidimetric assay from Randox Laboratories LtD, UK, on an Advia 1650.

EDTA plasma (K3-EDTA 1.6 mg/mL blood) for the separation of LDL subfractions was stored at -80°C. Plasma adjusted to a density of 1.067 g/L by iodixanol (Optiprep 60%, Axis-Schield PoC As, Oslo, Norway) was prestained with Coomassie blue, underlayered beneath 9% iodixanol and subjected to ultracentrifugation (2 1/2 h, 65,000 rpm 16ºC, (341,000 *g*) in a near vertical rotor (Beckmann NVT65). A digital photograph of LDL subclass profiles was analysed using Total Lab 1D gel-scan software (Pharmacis, UK). The LDL subfraction pattern was characterised based on the fractional (percentage) occurrence of small dense particles (density >1.031 g/mL) and large, buoyant particles (density <1.031 g/mL)[12]. The method has previously been described in detail [13,14].

The technique applied for CT CAG has previously been described by Achenbach et al [15]. The scans were conducted on a General Electric 64 slice scanner (LightSpeed VCT). The contrast agent was Jomeron^®^, 400 mg iod/mL. Scans were performed mainly as helical scans. Only arteries with an estimated luminal diameter >1.5 cm were examined, and stenoses were, as with CAG, considered significant if they obstructed at least 50% of the lumen. CAD was considered present if one or more vessels had a significant luminal obstruction.

Invasive CAG as well as analyses of blood samples were performed after the CT CAG examination and blinded to the evaluation of this.

CAG was conducted according to the routine protocol applied by our department and with standard equipment. Only arteries with an estimated luminal diameter >1.5 cm were examined, and stenoses were considered significant if they obstructed at least 50% of the lumen. CAD was considered present if one or more vessels had a significant luminal obstruction.

All participants gave an informed, written statement of consent. The study was approved by the regional ethical committee and conducted according to the Declaration of Helsinki.

### Statistics

All statistical analyses were conducted in "R", version 2.9.1. T-tests were used for comparisons of continuous data. Equality of variances was tested and allowed for in the t-tests, and Fisher's exact test was applied for comparisons of binary exposures. Parallel analyses were conducted based on CAG and CT CAG, respectively, i.e. the patients were stratified into "CAD" and "no CAD" according to either and then compared with respect to risk factors. To access the impact of sdLDL on presence of CAD binary, logistic regression analyses were made based on 10% increments in sdLDL, and a number of possible confounders, i.e. age, gender, current smoking, waist circumference, and LDL cholesterol, were interchangeably adjusted for. To ensure that no important differences were overlooked all data were analysed as median values as well and compared with the Wilcoxon rank sum test (data not shown).

## Results

### Invasive CAG

When stratified by invasive CAG (Table 2), patients with CAD were significantly more likely to be of male gender and to receive lipid-lowering drugs. They had a larger waist circumference, but no differences were observed as to age, family disposition, antihypertensive medication, BMI, current smoking, or blood pressure. Patients with CAD had significantly higher triglyceride and lower HDL cholesterol values, but did not significantly differ with respect to total cholesterol, LDL cholesterol, hsCRP, Lp(a), or apoB.

**Table 2:** Basic characteristics of patients, stratified according to presence of CAD verified by invasive CAG and CT CAG, respectively

	CAG			CT CAG		
Variable	No CAD (n = 120)	CAD (n = 58)	p-value	No CAD (n = 71)	CAD (n = 91)	p-value

Age (mean and SD)^T^	61.6 ± 9.8	64.1 ± 8.9	0.1	60.4 ± 9.8	64.1 ± 8.9	0.02

Male (number and %)^F^	46 (38.3)	42 (72.4)	<0.0001	40.8 (29)	57.1 (52)	0.06

Family disposition to ischemic heart disease (number and %)^F^	66 (55)	28 (48.3)	0.43	62.0 (44)	48.4 (44)	0.11

Actual smoking (number and %)^F^	21 (17.5)	17 (29.3)	0.08	19.7 (14)	24.2 (22)	0.57

Lipid-lowering medication (number and %)^F^	61 (50.8)	44 (75.9)	0.002	45.1 (32)	70.3 (64)	0.001

Antihypertensive medication (number and %)^F^	75 (62.5)	45 (77.6)	0.06	62.0 (44)	71.4 (65)	0.24

Systolic blood pressure (mean and SD)^T^	145.8 ± 21.6	148.6 ± 16.8	0.35	144.0 ± 20.1	147.4 ± 19.7	0.29

Diastolic blood pressure (mean and SD)^T^	78.8 ± 11.0	80.4 ± 9.2	0.34	78.2 ± 9.7	79.6 ± 10.6	0.39

BMI (kg/m^2^)(mean and SD)^T^	26.7 ± 4.3	27.2 ± 3.1	0.4	25.9 ± 4.0	27.4 ± 3.9	0.02

Waist circumference (cm) (mean and SD)^T^	94.8 ± 12.4	99.6 ± 9.7	0.005	92.8 ± 11.9	98.1 ± 11.4	<0.005

Total cholesterol (mmol/L) (mean and SD)^T^	5.0 ± 0.9	5.0 ± 1.0	0.75	5.1 ± 1.0	4.9 ± 0.9	0.35

*Triglycerides (mmol/L) (mean and SD)*^*T*^	*1.3* *0.7*	*1.8* *1.3*	*0.02*	*1.3* *0.7*	*1.2* *0.8*	*0.1*

*HDL cholesterol (mmol/L ) (mean and SD)*^*T*^	*1.7* *0.5*	*1.4* *0.4*	*<0.001*	*1.7* *0.5*	*1.5* *0.4*	*0.03*

LDL cholesterol (mmol/L) (mean and SD)^T ^(n_total _= 175)	2.7 ± 0.9 (n = 119)	2.8 ± 0.8 (n = 56)	0.39	2.8 ± 1.0 (n = 70)	2.7 ± 0.8 (n = 89)	0.46

*High sensitive CRP (mean and SD)*^*T*^	*2.2* *2.6*	*4.0* *7.2*	*0.06*	*2.2* *3.2*	*3.4* *5.8*	*0.09*

ApoB (g/L) (mean and SD)^T^	0.9 ± 0.2	1.0 ± 0.2	0.09	0.9 ± 0.2	0.9 ± 0.2	0.91

*Lp(a) (arb. units) (mean and SD)*^*T*^	*315.1* *387.9*	*456.4* *511.0*	*0.06*	*276.5* *346.2*	*412.9* *487.4*	*0.07*

sdLDL (mean and SD percentages)^T^	40.0 16.8	50.1 19.5	<0.001	39.5 17.1	45.8 18.7	0.03

Numbers in parentheses. (CAG: n = 178 and CT CAG: n = 162 ) (F: Fisher's exact test for count data, T: Students t-test, W: Wilcoxon rank sum test)

**Figure 1:**
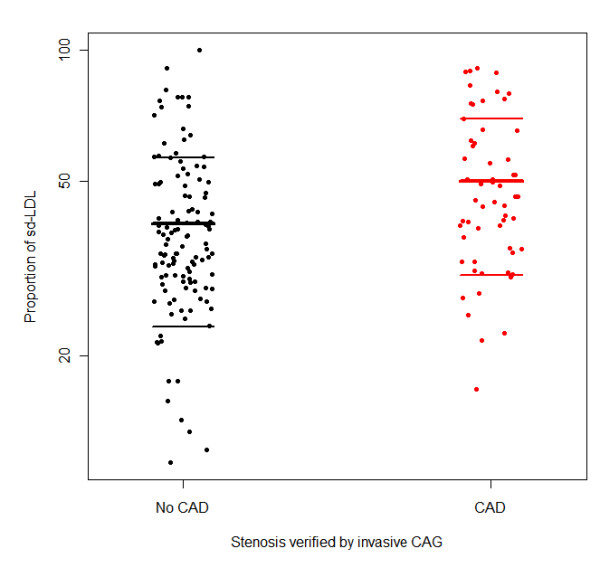
**Proportion of small dense LDL stratified according to presence of CAD verified by invasive CAG**. (n = 178; lines mean mean ± sd). The mean proportions were 40.0% and 50.1%, respectively.

As depicted in Figure 1, patients with CAD had a significantly higher proportion of small dense LDL (means 50.1% and 40.0%, respectively; p < 0.001).

Unadjusted sdLDL was a significant predictor of presence of CAD verified by invasive CAG in a logistic regression analysis (Table 3; Crude). Adjustment for age, gender, current smoking, and waist circumference (Adjustment 1) or adjustment for LDL cholesterol (Adjustment 2) did not materially reduce the estimate.

**Table 3:** Logistic regression analysis of CAD verified by invasive CAG with proportions of sdLDL as exposure (n = 175)

	Odds ratio	95% CI	P
Crude	1.36	1.13 1.64	0.001

Adjustment 1	1.26	1.02 1.56	<0.01

Adjustment 2	1.26	1.02 1.56	0.03

Odds ratios are expressed relative to sdLDL increments of 10%. (Adjusted 1: age, gender, current smoking, waist circumference; adjusted 2: age, gender, current smoking, waist circumference, LDL cholesterol)

### CT CAG

**Figure 2:**
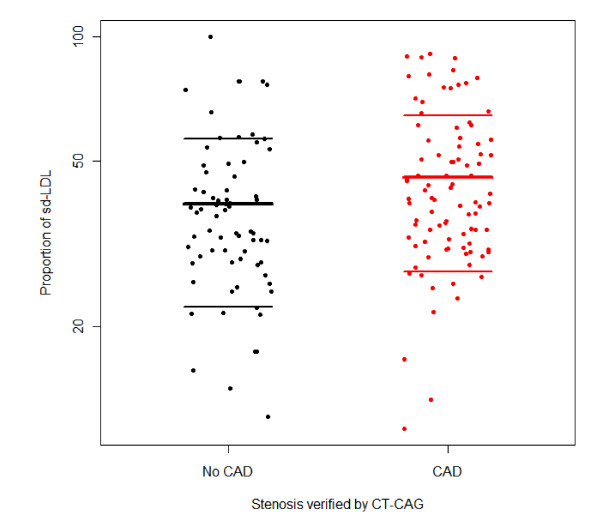
**Proportion of small dense LDL, stratified according to presence of CAD verified by CT CAG. (n = 162; lines mean mean ± sd)**. The proportions were 39.5% and 45.8%, respectively.

Stratified according to CT CAG, the risk profiles of the patients were slightly different (Table 2). Patients with CAD were older, more likely to receive lipid-lowering drugs, and had higher BMI and larger waist circumference. No lipid parameters, total cholesterol, LDL cholesterol, and triglycerides differed between patients with and without CAD, apart from patients with CAD having higher HDL cholesterol. Patients without and with CAD verified by CT CAG differed significantly in proportions of sdLDL (means 39.5% and 45.8%, respectively; p = 0.029) (Figure 2).

Crude sdLDL was a significant predictor of presence of CAD verified by invasive CAG in a logistic regression (Table 4; Crude). Adjustment for age, gender, current smoking, and waist circumference (Adjustment 1) reduced the estimate and rendered the prediction insignificant. Additional adjustment for LDL cholesterol (Adjustment 2) reduced the estimate and abolished the significance.

**Table 4:** Logistic regression analysis of CAD verified by CT CAG with proportions of sdLDL as exposure (n = 159)

	Odds ratio	95% CI	P
Crude	1.26	1.03 1.53	0.02

Adjusted 1	1.20	0.96 1.50	0.11

Adjusted 2	1.21	0.97 1.52	0.09

Odds-ratios are expressed relative to sd-LDL increments of 10%. (Adjusted 1: age, gender, current smoking, waist circumference; adjusted 2: age, gender, current smoking, waist circumference, LDL cholesterol)

## Discussion

The present study demonstrates that the proportion of small dense LDL particles is a strong univariate predictor of clinically significant coronary luminal stenosis and, in this data set, as strong a predictor as HDL cholesterol. In our linear models we did not adjust for HDL cholesterol and triglycerides, it being in our opinion meaningless, as the three parameters are intimately metabolically linked.

The importance of sdLDL in CAD, measured by different techniques, has been emphasised in several investigations, among those the Quebec Cardiovascular Study [16] in which men with an elevated sdLDL cholesterol had a significantly higher risk of coronary heart disease (CHD) on follow-up. Also in the EPIC-Norfolk study [17], patients with coronary disease presented with a smaller LDL peak particle size, a higher proportion of sdLDL, and an increased plasma concentration of sdLDL cholesterol than matched, healthy controls. Similar conclusions were drawn in a coinciding evaluation of the same study population by El Harchaoui [18] who reported a stronger association between the number of sdLDL particles and occurrence of CAD, than between serum LDL cholesterol and CAD. In accordance with this finding, we saw no significant difference in LDL cholesterol in groups with or without CAD, nor did adjustment for LDL cholesterol influence the relation between CAD and sdLDL particles, perhaps because the patients included in the study deviated from the background population by an a priori moderately altered risk profile and the use of statins.

Recognition of an intimate metabolic relationship between triglycerides and other lipids has fuelled debate as to which component is the principal marker of CVD risk and has given rise to the description of a lipid phenotype consisting of a high proportion of small dense LDL, high levels of triglycerides, and low concentrations of HDL cholesterol [7] typical of patients with the metabolic syndrome. Conversely, its degree of covariance with other risk factors may help explain why the strength of associations of these factors with CAD is diminished through statistical adjustment. In accordance with this, Austin et al. [19] found a significant, univariate negative correlation between LDL size and CAD that vanished upon adjustment for HDL cholesterol, triglycerides, and other traditional risk factors. In the Women's Health study [20], where LDL particle concentration was compared to LDL particle size as risk predictors of coronary mortality and morbidity, even the stronger of the two, the particle concentration, was strongly attenuated by adjustment. That sdLDL and LDL particle numbers are by no means independent parameters has been illustrated by Griffin et al. who reported that a predominance of sdLDL, moderately elevated triglycerides, and a low HDL cholesterol were all inversely associated with the number of LDL particles [13].

In a case-control study of 225 middle-aged Japanese CAD patients [21], Koba et al. found that the coronary stenosis was highly correlated with an LDL particle diameter less than 255 Å. The CAD patients had LDL cholesterol and non-HDL cholesterol similar to healthy controls, but sdLDL cholesterol was elevated. In a logistic regression analysis, adjustment for HDL cholesterol did, however, not abolish the effect of sdLDL cholesterol. The present study differs from that study mainly in the measurement of the "sd" parameter (Kola et al. measured cholesterol content whereas we measured the sdLDL proportion) and in the recruitment of patients. Our patients were enrolled based on a clinical suspicion of CAD prior to enrolment, whereas in the study from Japan, confirmed CAD patients were compared to healthy controls who were younger and had a healthier life style.

Kwon et al. [22] used a cross-sectional design to investigate the relation of LDL particle size to CAD. The study population consisted of 504 patients without a prior history of myocardial infarction who underwent a CAG for evaluation of chest pain. Among these, 262 had at least one stenosis by CAG that obstructed 50% of the lumen, while 242 patients, who showed no or only minimal signs of CAD on a subsequent angiographic examination, served as controls. In accordance with our study, the fraction of sdLDL was significantly higher in the CAD group. In contrast to our data, however, sdLDL remained a significant risk factor upon adjustment for traditional risk factors, HDL cholesterol, and LDL cholesterol. Kwon et al. used electrophoresis yielding a size scale with a specific nanometre cut-off point between small and large LDL particles, whereas our measurements were based on density and a density cut-off. Indeed, several reports, among those a systematic review [23], emphasise the incongruence between techniques.

Finally, Koz et al. [24] conducted a study in 102 consecutive young men with chest pain and a low Framingham risk score. They all underwent a CAG. LDL size was smaller in patients with CAD (n = 45), but it was not a significant predictor upon adjustment for traditional risk factors. The results are therefore in good agreement with ours. Moreover, the fact that the study populations were quite dissimilar from ours (young men performing military service vs elderly patients) emphasises the significance of the sdLDL parameter.

The alternative grouping of patients based on CT CAG in our study in general showed a weaker correlation with sdLDL, and its predictive value was lost after adjustment for traditional risk parameters alone. This could in part be explained by a reduced sample size, but it might also reflect the fact that more participants in our study were classified as having CAD on CT examination than on invasive CAG. CT CAG is a fast emerging image modality, but the specificity of the method is suboptimal, and this might readily explain the weaker correlation between sdLDL and CT CAG-evaluated CAD.

The present study has some limitations. The sample size was relatively small, and a priori patients belonged to the low and intermediate CAD risk groups. Likewise, the number of patients with significant stenoses was too limited to enable us to graduate the extent rather than the mere presence of CAD. Identifying patients with CAD on the basis of significant stenoses may underestimate the total number of patients with CAD, as visually significant stenoses only represent patients with advanced disease. Treatment with statins and β-adrenoceptor antagonists is widespread in Denmark. Apart from a reduction of LDL cholesterol and varying effect on other lipid parameters dependent on the type of dyslipidemia and the specific agent, statins have been shown to induce a significant shift in the sdLDL subtype pattern towards larger and more buoyant particles [25]. Thus, as a larger percentage of the CAD group than of the no CAD group received statins, the connection between sdLDL and CAD may be even stronger than depicted here.

The findings of the present study provide further support to the value of not only cholesterol in the major lipoprotein subfractions (HDL and LDL), but also the distribution and quality of particles within each subfraction in risk assessment, as sdLDL, in contrast to LDL cholesterol, emerged as a univariate predictor of CAD. HDL cholesterol was, statistically, as closely correlated to the CAD status, but the absolute difference between the mean HDL values in the two groups was small compared to the differences of sdLDL. The study also demonstrates the clinical utility of the iodixanol method which, in contrast to the expensive NMR analysis, can be routinely incorporated into clinical risk assessment. However, the widespread use of statins makes it impossible to exclude patients receiving this treatment and to generalize data to other populations.

## Conclusion

A significant difference in the proportion of sdLDL was observed between patients with CAD and otherwise highly similar patients without CAD. Clinical trials are necessary to determine whether this is of importance for stratification or patient outcome.

## Abbreviations

CAD: coronary Artery Disease; CAG: invasive coronary angiography; CHD: coronary heart disease; CT: CAG CT-based coronary angiography; HDL: high-density lipoprotein; LDL: low-density lipoprotein; PCI: percutaneous coronary intervention; sdLDL: small dense LDL particles

## Competing interests

The authors declare that they have no competing interests.

## Authors' contributions

APTP compiled the data and wrote the first draft of the paper. HHT and KR evaluated the CT scans (blinded to the invasive coronary angiography results). JA evaluated the results from invasive coronary angiography (blinded to the results of the CT scans). THC was responsible for the technical aspects of the CT scans and helped with the interpretation of the results. BAG set up the analysis of small dense LDL cholesterol particles and interpreted the results together with IVA and AA. HHT, KR, JA, and EBS developed the scientific idea behind the study. All authors contributed to a critical assessment of the manuscript and read and approved the final version.
